# The Book World of Medicine and Science

**Published:** 1900-11-24

**Authors:** 


### The Book World of Medicine and Science.

The Student's Medical Dictionary, including all the Words and Phrases generally used in Medicine, with their proper Pronunciation and Definition. By George M. Gould, A.M., M.D., with a new Table of Eponymic Terms and Tests. Eleventh Edition, enlarged, with many illustrations. (London: H. K. Lewis. 1900. Price 14s. net.)

This work is intended for the use of those engaged in the study of medicine, dentistry, and pharmacy, or in the early years of practice, and, as is stated in the preface, cannot be used as a substitute for the larger dictionary by the same author, but rather as an introduction to it. Nevertheless, we think that many people will find in it all they want, seeing that it gives the derivation, the pronunciation, and a more or less complete definition of the vast majority of the words ordinarily met with in medical works, the meaning being in many cases rendered clear by woodcuts in the text.

It is, perhaps, unfortunate for the future of the English language that the Americans should have shown themselves such adepts at dictionary making, for by virtue of their efforts in this direction many forms of spelling which might otherwise have proved but temporary curiosities have obtained a sort of sanction which it is becoming very difficult to contend against. Fiber, fetus, center, anemia, hyperemia, leucorrhea, metrorrhea, edema, may do well in America, but such words seem unfamiliar to English eyes. Fashion in chemical nomenclature changes so rapidly that we do not like to speak positively about oxids and chlorids and sulphids, but the ruthless elimination of the final "e" gives these words a strange appearance. Unfortunately for the uniformity of his dictionary, Dr. Gould has not rooted out all his diphthongs with equal vigour. Thus although anæmia is spelled "anemia," in anæsthesia one is allowed a choice of using the diphthong or not. In some places, moreover, where he has knocked out the words containing the double letters he has not inserted the substitute; thus under "Cœlom" we are given no definition, but told to "see Celom." No such word, however, as "Celom" is to be found. Again, under the word "Cæcal" we are told, "See Cecal," under "Cæcum" "See Cecum," under "Cæsitis" "See Cecitis." But there is neither Cecum, Cecal, nor Cesitis in the dictionary—which is bad. If the language is to be twisted about in this way the work ought at least to be done with some attempt at uniformity and completeness. In the word "Celiotomy" we are given no alternative, while in "Celiitis, Cæliitis," we are offered a choice of spellings. So much for criticism. On the other hand there is a great deal in this dictionary which is very good, and the Table of Eponymic Terms will be found distinctly useful. Without some such reminder who is to remember the meaning of such terms as "Agostini's Reaction," "Ahlfield's Sign," "Albert's Disease," "Albini's Nodules" "Allen's Reaction," "Allingham's Painful Ulcer," "Adamkiewicz's Reaction," a small selection from the first page of this appendix?

Malaria according to the New Researches. By Professor Angelo Celli, Director of the Institute of Hygiene, University of Rome. Translated by John Joseph Eyre, M.R.C.P., L.R.C.S. Irel., D.P.H. Camb. With an introduction by Dr. Patrick Manson, Medical Advisor to the Colonial Office. Maps and Illustrations. (Longmans, Green and Co. 1900. Price 10s. 6d.)

Both as to the original work and its translation this is a book to which we may honestly accord the highest praise. It is indeed seldom that we meet with so complete a presentment of a great subject, containing so little that is extraneous, and yet showing so well, as does this work of Celli's, how wide and far-reaching are the influence and importance of the matter dealt with. The book is divided into two portions, the first being on Epidemiology, the second on Prophylaxis. In the first part we find an admirable description of the ætiology of malaria as it occurs, not only in man, but in various other animals; of the sources of infection of the characters and habits of the malarial mosquitoes; and of the mode in which the germ of the disease enters the body; in fact we have a good and well-reasoned account of the mosquito theory of malaria as it is now generally accepted. There is much, however, besides this, and many people will, we think, find the most interesting parts of this book, as distinct from other works on the subject which have recently been published, to lie in the full descriptions given of the circumstances in which malaria, as it is seen in Italy, mostly occurs; of the extent of its ravages; of the social conditions which, while in part caused by the prevalence of the disease, are also direcly provocative of its continuance; and of the conditions and surroundings which experience has shown to tend to the establishment of some sort of immunity to it. The history of the disease is interesting as showing for how far back malaria has been present in one part or another of Italy, and how immense has been and still is the injury inflicted on that country by this scourge. When we are told that the mean mortality from malaria in Italy is about 15,000 victims per annum, and that (it being a disease for which we have an efficacious specific remedy) its fatality is low, namely, about 7·75 per 1,000 of those who suffer from it, we may recognise the extent of the epidemy. If, then, we consider that there are, approximately, two million cases a year, and that the duration of a malarial infection is generally long, we begin to see the enormous loss in labour and in production, beside the loss of life, that Italy suffers from the prevalence of this disease. In the portion of the book devoted to prophylaxis an interesting review is given of the various expedients which have been from time to time proposed for the prevention or mitigation of the infection, and it is made evident that the suppression of malaria is not a matter of one measure, but of many. The greatness of the task is only equalled by its importance; but, at any rate, in temperate climates, where a considerable period of time must intervene between successive malarial seasons, it ought not to be impossible to put a limit to this pest. We wish we could speak as hopefully in regard to countries in which the climate is such as to enable the parasite to develop within the body of the mosquito all the year round. To suppress malaria in such places will be a task indeed. The book is an admirable study of the whole question as its exists in Italy, and it concludes with a full bibliography of Roman malaria, which will be found useful by those who wish to examine the question more minutely.

